# Production, Purification, and Characterization of Polygalacturonase from *Mucor circinelloides* ITCC 6025

**DOI:** 10.4061/2010/170549

**Published:** 2010-06-01

**Authors:** Akhilesh Thakur, Roma Pahwa, Smarika Singh, Reena Gupta

**Affiliations:** Deparment of Biotechnology, Himachal Pradesh University, Summer Hill, Shimla 171005, India

## Abstract

*Mucor circinelloides* produced an extracellular polygalacturonase enzyme, the production of which was enhanced when various production parameters were optimized. Maximum polygalacturonase (PGase) activity was obtained in 48 h at 30°C and pH 4.0 with pectin methyl ester (1% w/v) as carbon source and a combination of casein hydrolysate (0.1% w/v) and yeast extract (0.1% w/v) as nitrogen source. The enzyme was purified to homogeneity (13.3-fold) by Sephacryl S-100 gel-filtration chromatography. Its molecular weight was 66 kDa on SDS-PAGE. The enzyme was found to have *K*_*m*_ and *V*_max_ values of 2.2 mM and 4.81 IU/ml at 0.1% to 0.5% (w/v) concentration of the substrate. The addition of phenolic acids (0.05 mM), metal ions such as Mn^+2^, Co^+2^, Mg^+2^, Fe^+3^, Al^+3^, Hg^+2^, and Cu^+2^, and thiols had inhibitory effect on the enzyme. The enzyme showed maximum activity in the presence of polygalacturonic acid (0.1% w/v) at pH 5.5 and 42°C.

## 1. Introduction

Pectinases hydrolyze pectic substances. They have a share of 25% in the global sale of the food enzymes. Pectinases are one of the most widely distributed enzymes in bacteria, fungi, and plants [1]. Polygalacturonases (PGases) catalyze the random hydrolysis of 1,4 *α*-D galacturonic acid linkages in smooth region of pectin [2, 3]. The pectinolytic enzymes degrade the pectic substances found in plant tissues, thereby, having numerous applications in various types of industries such as juice and food industries [4], for example, increase in the yield of fruit juice from pulp, removal of haze from juices to get a clear product [5], paper and pulp industry [6], natural fibres treatment in textile industries [7] such as retting of flax fibres [8], production of Japanese paper [9], and bast fibers [10]. Pectinase production by microbes varies according to the composition of growth medium and the cultivation conditions, that is, pH, temperature, aeration, agitation, and incubation time [11]. Solid state fermentation has been found to be more suitable for pectinase production as compared to the submerged condition [12]. Recently, five pectinolytic enzymes by *Aspergillus niger* MIUG-16 were purified using a combination of chromatographic techniques [13]. Commercial enzyme preparations used in processing of food, traditionally, comprising of the mixtures of polygalacturonase, pectate lyase, and pectin esterase, are almost exclusively derived from fungal species, especially *Mucor* and *Aspergillus *[14]. Biochemical and thermal characterization of crude exo-polygalacturonase produced by *Aspergillus sojae* has also been reported [15]. Recently, the enzyme polygalacturonase has been targeted to develop nonfungicidal control strategies in order to avoid postharvest losses due to a fungal pathogen [16]. Each application requires unique properties with respect to specificity, stability, temperature, and pH dependence. The immense potential of polygalacturonase, particularly its application in juice clarification, can be of tremendous importance to Himachal Pradesh as it deals with large amount of horticultural products, especially apples. The apple juice faces the problems of turbidity and suspended particles like pectin, cellulose, and hemicellulose. Due to these factors, the juice fails to match international standards. The quality of juice can be improved by appropriate treatment with pectinolytic enzymes leading to the increased market potential, thereby, providing a significant boost to the economy of hill state. 

Keeping in view the above consideration, in this paper we report production, purification, and characterization of PGase from saprophytic fungus, *Mucor circinelloides* ITCC 6025.

## 2. Materials and Methods

### 2.1. Chemicals

All the chemicals used were either procured from Sigma Aldrich (USA) or HIMEDIA (Mumbai, India) and were of high analytical grade. Polygalacturonic acid from citrus fruit (Sigma) was used as substrate. The fungus *Mucor circinelloides *ITCC 6025 was isolated from soil and identified at the division of plant pathology, IARI, New Delhi. 

### 2.2. Maintenance of *M. circinelloides* and Polygalacturonase Production

The fungal culture was stored on Potato Dextrose Agar (PDA) slants and incubated at 30°C for 5 days. The heavily sporulated slants were stored at 4°C and subculturing was done after every 45 days. The spores were harvested by adding 2 mL sterile saline containing 0.01% Tween 20 to a sporulated slant. About 2 × 10^5^ spores were required for better production of enzyme. 500 mL of medium consisting of KCl (0.05%), MgSO_4_.7H_2_O (0.01%), trisodium citrate dihydrate (0.1%), Citric acid anhydrous (0.1%), Yeast extract (0.1%), Casein hydrolysate (0.1%), and Pectin (1%) was used for production purpose. The pH of the medium was adjusted to 4.0 with 0.1 N HCl. The medium was autoclaved at 15 psi for 20 minutes. 25 mL of sterile production medium was taken in 250 mL conical flasks, or 50 mL of sterile production medium was taken in 500 mL conical flasks for better growth and production. About 2 × 10^5^ spores were inoculated in each 250 mL conical flask and the flasks were incubated in an ORBITEK rotary shaker at 30°C at 150 rpm. After 48 hours, the culture was harvested and filtered through Whatmann no. 1 filter paper. The filtered cell-free broth was used as crude enzyme. The effect of various carbon sources, that is, pectin (methyl ester), apple pectin, glucose, fructose, galactose, and maltose (1% w/v) was studied at pH 4.0 and temperature 30°C. Each of various nitrogen sources ammonium sulphate, ammonium nitrate, ammonium chloride, ammonium dihydrogen phosphate, casein hydrolysate, and yeast extract was individually added to production broth at a concentration of 0.1% (w/v).

### 2.3. Enzyme Assay

The PGase activity was assayed by estimating the amount of reducing sugar released under assay conditions. The reducing sugar was quantified by the method of Somogyi [17]. The substrate used for assay was 0.5% PGA (polygalacturonic acid), that is, 0.5 g of PGA in 100 mL of 0.05 M citric acid-sodium citrate buffer, pH 5.4. The assay mixture consisted of 980 *μ*l of freshly prepared substrate and 20 *μ*l of enzyme and incubated at 40°C for 20 minutes. The reaction was stopped by keeping the reaction mixture in boiling water bath for 3 minutes. To the reaction mixture, 500 *μ*l of alkaline copper tartrate reagent was added and tubes were again kept in boiling water bath for 20 minutes. In the control, 20 *μ*l of enzyme was added after adding alkaline copper tartrate reagent. The tubes were cooled and 500 *μ*l of arsenomolybdate reagent was added and thoroughly mixed. The absorbance was recorded at 620 nm (using Perkin Elmer model Lambda 12 UV/VIS spectrometer) against a blank consisting of 1 mL citrate buffer, 500 *μ*l alkaline copper tartrate reagent, and 500 *μ*l arsenomolybdate reagent. The amount of galacturonic acid released per mL per minute was calculated from standard curve of galacturonic acid. One Unit of PGase activity was defined as the amount of enzyme required to release one micromole of galacturonic acid per mL per minute under standard assay conditions.

### 2.4. Determination of Protein Concentration

The protein concentration was determined using BSA as standard by the method of Lowry et al. [18].

### 2.5. Purification by Gel-Filtration (Sephacryl S-100) Chromatography

PGase was purified to homogeneity by chilled ethanol (60%) precipitation and column chromatography. The partial purification of enzyme was done by adding chilled ethanol (60%) to crude enzyme and keeping it for overnight incubation. The precipitates thus obtained were spun at 15000 rpm for 15 minutes at 4°C. The pellet obtained was dissolved in minimum volume of assay buffer (sodium-citrate buffer), pH 5.4. It was again centrifuged at 25000 rpm for 20 minutes at 4°C to obtain a viscous sample. The precipitated enzyme was filtered through 0.22 *μ* filter, and 2 mL of this filtered precipitated enzyme was loaded on gel-permeation column Sephacryl S-100 (bed volume 120 mL). All the eluted fractions were estimated for enzyme activity and absorbance at 280 nm. The fractions showing the highest PGase activity were pooled and assayed for protein content. The specific activity of purified enzyme fractions was compared to that of crude enzyme and fold purification was calculated. Purified enzyme was lyophilized by Flexi-Dry MP to make it more concentrated for SDS-PAGE.

The relative molecular weight of the purified enzyme was estimated by SDS-PAGE (12%) according to the method of Laemmli [19]. Proteins were stained with coomassie brilliant blue R-250.

### 2.6. Characterization of Polygalacturonase

#### 2.6.1. Optimization of Temperature and pH

The optimum temperature and pH of the enzyme were determined by measuring the PGase activity at various temperatures (36, 38, 40, 42, 44, and 46°C) at pH 5.4 and pH (4.0, 4.5, 5.0, 5.5, 6.0, and 6.5) at 42°C using 50 mM citrate buffer with PGA (0.5%) as substrate. 

#### 2.6.2. Optimization of Incubation Time

Optimum reaction time was determined by incubating the reactants for different time intervals (5, 10, 15, 20, 25, and 30 minutes) and performing the assay for PGase activity at optimized temperature and pH.

#### 2.6.3. pH Stability of Enzyme

To determine the pH stability, the enzyme was preincubated in 0.05 mM sodium-citrate buffer (pH 3.5–7.0) for 4 hours at 42°C and the enzyme activity was assayed under standard assay conditions.

#### 2.6.4. Thermostability of Enzyme

To determine the half-life of enzyme, the enzyme was incubated at 42°C and the enzyme activity was determined upto 7 hours after every 1 hour. For determination of thermostability, the enzyme was incubated at 50°C for 5 hours and the enzyme activity was determined after every 30 minutes.

#### 2.6.5. Effect of Metal Ions

The assay was performed in the presence of various metal ions at a final concentration of 1 mM in 50 mM sodium-citrate buffer pH 5.4.

#### 2.6.6. Substrate Specificity

A study of substrate specificity for polygalacturonase from **M. circinelloides** was made by using polygalacturonic acid, pectin citrus (DE-89%), pectin apple (methyl 7.8%), potato dextrose agar, and amylopectin (0.1% each).

#### 2.6.7. Determination of *K*_*m*_ and *V*_*max*_ Value

*K*_*m*_ and *V*_max _ values of the enzyme were determined by measuring the reaction velocity at different concentrations of the substrate (PGA) 0.1% to 0.5% (w/v). The reciprocal of the reaction velocity (1/V) was plotted against the reciprocal of the substrate concentration (1/[S]) to determine the *K*_*m*_ and *V*_max _ values by the Line-Weaver-Burke plot.

#### 2.6.8. Effect of Thiols and Reducing Agents

A study of different thiols was performed by preincubating the enzyme (0.3 mL) for 5 minutes at 37°C before starting the reaction with PGA making the final concentration of thiols to 1 mM with 0.05 M citrate buffer, pH 5.4.

#### 2.6.9. Effect of Phenolics

The assay was performed in the presence of various phenolic acids at a final concentration of 0.05 mM in 50 mM citrate buffer, pH 5.4, by preincubating the enzyme for 5 minutes at 37°C before starting the reaction with PGA. 

## 3. Results and Discussion

### 3.1. Polygalacturonase (PGase) Production by *M. circinelloides* ITCC-6025

Amongst various carbon sources tested for PGase production pectin methyl ester 1% (w/v) was the best source followed by apple pectin (Table 1). Earlier, the PGase activity was reported to be maximally supported by pure pectin followed by wheat bran and maximum PGase activity was observed in citrus pectin (Var. mussami) by thermophilic *Aspergillus fumigatus* [20]. PGase production was enhanced using various sugars and complex agro-products 1% (w/v) [21]. Addition of glucose in culture medium led to decrease in PGase production in *Aspergillus niger *[22]. 

Amongst various nitrogen sources tested, the best PGase production was obtained when Casein hydrolysate (CH) 0.1% (w/v) and Yeast extract (YE) 0.1% (w/v) were used together. YE alone also gave comparable activity. Among the inorganic nitrogen sources, maximum activity was obtained with NH_4_Cl (Table 2). There have been reports of enhanced PGase production when NH_4_Cl was used as nitrogen source [23]. It has been reported that nitrogen limitation decreases the polygalacturonase production [24]. 

### 3.2. Enzyme Purification

**Figure 1:**
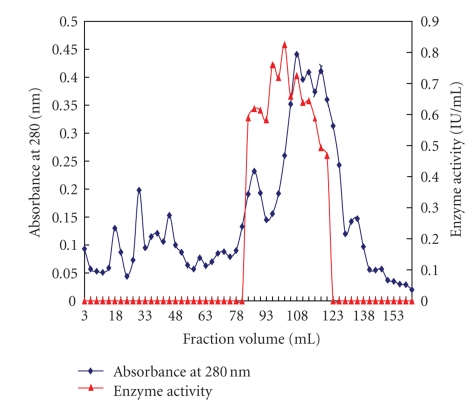
Elution profile of polygalacturonase on Sephacryl S-100 column.

The enzyme was purified about 13.3-fold with a specific activity of 31.74 IU/mg giving a yield of 3.4% after Sephacryl S-100 gel-permeation chromatography (Figure 1, Table 3) which resulted in almost a single peak when absorbance was recorded at 280 nm. Earlier, various polygalacturonases have been purified using gel-permeation chromatography [25, 26]. 

**Figure 2:**
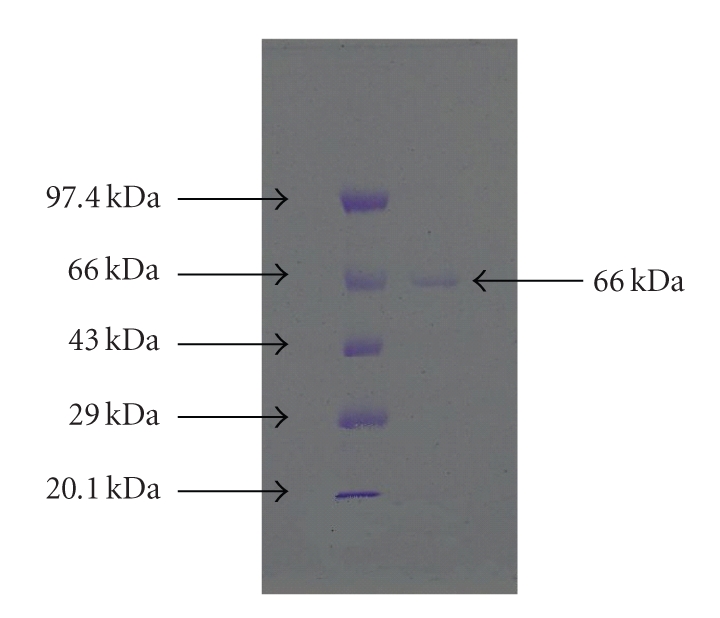
SDS-PAGE of purified polygalacturonase from *Mucor circinelloides*. Lane 1: Bangalore Genei Protein marker ( kDa). Phosphorylase b 97.4. Bovine Serum Albumin 66.0. Ovalbumin 43.0. Carbonic anhydrase 29.0. Soybean Trypsin Inhibitor 20.1. Lane 2: Lyophilized concentrated fraction from Sephacryl S-100 Column.

The purified polygalacturonase showed a single band on 12% SDS-PAGE (Figure 2). The molecular weight was found to be 66 kDa which indicated that it was novel enzyme from *Mucor *sp. It was comparable to exoenzymes secreted by some other fungi [27–30]. 

### 3.3. Enzyme Characterization

**Figure 3:**
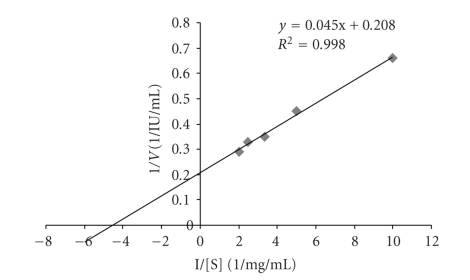
Line-weaver-Burke plot for polygalacturonase.

The purified enzyme exhibited optimum polygalacturonase activity at a temperature of 42°C (Table 4) and pH of 5.5 (Table 5). The maximum activity of polygalacturonase from **M. circinelloides** was obtained using 20 *μ*l of enzyme after 20 minutes incubation at 42°C and pH of 5.5 (Table 6). The enzyme was stable within pH range of 4.5 to 6.5 with optimum pH of 5.5. A further increase in pH led to a marked decrease in stability of the enzyme (Table 7). The enzyme was found to have a half-life of 5 hours at 42°C (Table 8) and 2 hours at 50°C (Table 9). Temperature optimum of 40°C or higher for polygalacturonase stability has been reported in banana fruits and psychrophilic fungus *Sclerotinia borealis* [31, 32]. Polygalacturonase from *M. flavus* has been reported to be optimally active between pH range 3.5 to 5.5 and stable up to 40°C for 4 hours [33]. The optimum pH of 7.0 has been reported for polygalacturonase from *Sporotrichum thermophile Apinis *[34]. The *K*_*m*_ and *V*_max _ values of polygalacturonase were found to be 2.2 mM and 4.81 IU/mL, respectively, at different concentrations of the substrate, that is, 0.1% to 0.5% (w/v) by plotting the Line-Weaver Burke plot (Figure 3). Exo-polygalacturonase from *Bacillus species* KSMP 443 [35] and *Penicillium frequentans* [36] were found to have comparable *K*_*m*_ values. 

Polygalacturonase showed 5–15% decrease in enzyme activity in presence of Mn^+2^, Co^+2^, and Mg^+2^ whereas Fe^+3^, Zn^+2^ caused 27–31% decrease in the enzyme activity (Table 10). Thus, the enzyme did not require any metal ions to express its activity. Earlier, the effect of different metal cations at the concentration of 1 mM on *T. harzianum* showed that all metal cations exhibited different and partial inhibitory effects on the activity of enzyme except Mn^+2^ and Co^+2^ which completely inhibited the enzyme activity [37]. Also, the addition of 0.01 mM HgCl_2_ increased the PG II activity of *A. niger* 3.4 times but did not affect PG I [38]. The purified polygalacturonase from **M. circinelloides** ITCC 6025 showed maximum activity with PGA (0.1% w/v), but it decreased with all other substrates indicating that PGA is the best substrate for maximum enzyme activity (Table 11). Polygalacturonase activity was progressively inhibited in the presence of thiols in comparison to that of control. Only L-cystine activated the reaction at 1mM concentration and showed enzyme activity 3.8 IU/mL in comparison to 3.5 IU/mL of control (Table 12). Mercuric chloride was the strongest inhibitor of enzyme at 1 mM concentration followed by ascorbic acid. Phenolic acids, namely, cinnamic acid, chlorogenic acid, p-coumaric acid, ferulic acid, and 2,4-dinitro salicylic acid inhibited the enzyme activity. Cinnamic acid showed maximum inhibition of enzyme activity followed by 2,4-dinitrosalicyclic acid (Table 13). Previously, comparable results have been obtained for the effect of thiols and phenolics [39].

Thus, polygalacturonase from **M. circinelloides** ITCC 6025 can be exposed for its potential application such as juice clarification, textile, plant fiber processing, tea, coffee, oil extraction, and treatment of industrial waste water containing pectinaceous material. In the present investigation, although polygalacturonase from **M. circinelloides** ITCC 6025 has effectively been purified and characterized, the enzyme properties may, however, be further improved via efficient immobilization onto a suitable matrix. The immobilized enzyme can be of industrial advantage in terms of sturdiness, availability, inertness, low price, reusability, and temperature stability [40]. 

## Figures and Tables

**Table 1:** Effect of various carbon sources on PGase production from *Mucor circinelloides. *

Carbon source 1% (w/v)	Enzyme activity (*μ*mol mL^−1^min^−1^)	Relative Activity (%)
Pectin (methyl ester)	9.15	100.00
Apple pectin	8.41	91.91
Glucose	2.12	23.16
Fructose	5.86	64.04
Galactose	2.30	25.13
Maltose	2.50	27.32

The experiment was carried out using casein hydrolysate as nitrogen source.

**Table 2:** Effect of various nitrogen sources on PGase production from *Mucor circinelloides. *

Nitrogen source 0.1% (w/v)	Enzyme activity (*μ*mol mL^−1^min^−1^)	Relative Activity (%)
(NH_4_)_2_SO_4_	4.56	45.64
(NH_4_)NO_3_	5.43	55.35
NH_4_Cl	6.25	62.56
(NH_4_)_2_SO_4_FeSO_4_	2.12	21.22
(NH_4_)H_2_PO_4_	3.16	31.63
CH	8.55	85.58
YE	9.55	99.59
CH+YE (Control)	9.99	100.00

The experiment was carried out using pectin methyl ester as carbon source.

**Table 3:** Purification of polygalacturonase produced extracellularly by *Mucor circinelloides. *

Step	Volume (mL)	Total Activity (IU)	Total Protein (mg)	Specific Activity (IU/mg)	Fold Purification	% Yield
Crude	1000	624	261	2.391	1	100
Ethanol (60%)	75	39.6	7.8	5.077	2.12	6.35
Sephacryl S-100	42	21.33	0.672	31.74	13.3	3.4

**Table 4:** Effect of temperature on activity of polygalacturonase from *Mucor circinelloides. *

Temperature (°C)	Activity (IU/mL)
36	1.58
38	2.32
40	2.75
42	3.80
44	2.40
46	1.55

The experiment was carried out at pH 5.4.

**Table 5:** Effect of pH on activity of polygalacturonase from *Mucor circinelloides. *

pH	Activity (IU/mL)
4.0	1.55
4.5	2.30
5.0	2.80
5.5	3.62
6.0	1.75
6.5	1.15

The experiment was carried out at a temperature of 42°C.

**Table 6:** Effect of reaction time on activity of polygalacturonase from *Mucor circinelloides. *

Reaction time (min.)	Activity (IU/mL)
5	1.80
10	2.80
15	3.45
20	3.82
25	1.82
30	1.10

The experiment was carried out at a temperature of 42°C and pH 5.5.

**Table 7:** Effect of pH on stability of polygalacturonase from *Mucor circinelloides. *

pH	Enzyme Activity (IU/mL)	Relative Activity (%)
3.5	1.50	41.0
4.0	2.30	63.0
4.5	2.51	68.7
5.0	3.42	93.6
5.5	3.65	100.0
6.0	2.68	73.4
6.5	2.45	67.1
7.0	1.30	35.6

**Table 8:** Stability of polygalacturonase at 42°C from *Mucor circinelloides. *

Time (h)	Enzyme Activity (IU/mL)	Relative Activity (%)
0	3.5	100.0
1	3.4	97.1
2	2.5	71.4
3	2.1	60.0
4	1.9	54.2
5	1.7	50.0
6	1.6	45.7
7	1.5	42.8

**Table 9:** Stability of polygalacturonase at 50°C from *Mucor circinelloides. *

Time (h)	Enzyme Activity (IU/mL)	Relative Activity (%)
0.0	3.70	100.00
0.5	3.60	97.20
1.0	2.70	72.90
1.5	2.20	59.45
2.0	1.81	50.00
2.5	1.65	44.50
3.0	1.34	36.20
3.5	1.25	33.70
4.0	1.10	29.70
4.5	0.92	24.80
5.0	0.85	22.90

**Table 10:** Effect of metal ions on activity of polygalacturonase from *Mucor circinelloides. *

Metal ions (1 mM)	Enzyme Activity (IU/mL)	Relative Activity (%)
Al^+3^	3.06	80.60
Fe^+2^	2.73	72.00
Hg^+2^	1.71	45.10
Mg^+2^	3.23	85.16
Mn^+2^	3.68	96.85
Zn^+2^	2.77	72.98
Co^+2^	3.13	82.60
Cu^+2^	3.07	80.80
Control	3.80	100.00

The experiment was carried out at a temperature of 42°C and pH 5.5.

**Table 11:** Effect of substrates on activity of polygalacturonase from *Mucor circinelloides. *

Substrate (0.1%)	Enzyme Activity (IU/mL)	Relative Activity (%)
PGA	3.50	100.0
Amylopectin	0.21	6.0
Potato Dextrose Agar	0.12	3.7
Pectin Citrus (DE-85%)	0.38	11.0
Pectin Apple (methyl-7.8%)	0.77	22.0

The experiment was carried out at a temperature of 42°C and pH 5.5.

**Table 12:** Effect of thiols on activity of polygalacturonase from *Mucor circinelloides. *

Thiols (1 mM)	Enzyme Activity (IU/mL)	Relative Activity (%)
L-Cystine	3.80	108.57
Ascorbic acid	0.52	14.85
B-Mercaptoethanol	2.48	70.80
Mercuric chloride	0.48	13.71
Sodium metabisulphite	1.20	34.00
Control	3.50	100.00

**Table 13:** Effect of phenolics on activity of polygalacturonase from *Mucor circinelloides. *

Phenolics (0.05 mM)	Enzyme Activity (IU/mL)	Relative Activity (%)
Cinnamic Acid	1.54	44.0
Chlorogenic Acid	2.05	58.0
p-Coumaric Acid	1.89	54.0
Ferulic Acid	1.96	56.0
2,4-dinitrosalicylic Acid	1.79	51.1
Control	3.51	100.0
